# The American Academy of Medicine; Tenth Annual Meeting

**Published:** 1886-11

**Authors:** 


THE AMERICAN ACADEMY OF MEDICINE

*Held its Tenth Annual Session at Pittsburg, Pa., on October 12 and 13, 1886.*—The meeting was called to order by DR. R. STANSBURY SUTTON, the President.

After considerable routine business, a paper was read by R. J. Dunglison, A.M., M.D., of Philadelphia, on "The Laws Regulating Practice in the Different States."

F. N. Gerrish, A.M., M.D., of Portland, Maine, followed with a paper. Subject, "The Best Equipment for Medical Study."

The special committee appointed to report on the best preliminary education for medical students next made its report. The chairman, F. N. Gerrish, A.M., M.D., spoke strongly in favor of a classical education for all students, and referred with feeling to the prostitution of the medical profession by colleges which were little more than mere speculators.

John D. Kelley, A.M., M.D., of Lowville, N. Y., presented a report on "The Status of Medical Education in the United States."

L. P. Bush, A.M., M.D., of Wilmington, Delaware, read a paper on "The Advantages of a Classical Education to the Medical Student and Physician." He took decided ground in favor of a classical education. W. H. Pancoast, A.M., M.D., of Philadelphia, thought that too much Latin and Greek were studied at a tender age. He thought youths should study more French and German. He referred to the German system of education, and said they are the most classical students in the world.

J. Cheston Morris, A.M., M.D., of Philadelphia, next read on "Prolonged Gestation," relating interesting cases.

A paper by Traill Green, A.M., M.D., Easton, Pa., "The Duties of Physicians to Each Other," was deferred to the annual meeting next year.

A. C. Kemper, A.M., M.D., Cincinnati, presented a paper entitled "A Christian Education the Best Preparation for the Practice of Medicine," which was read by the Secretary.

Henry O. Marcy, A.M., M.D., of Boston, presented a paper asking, "Is Modern Wound-Treatment Scientific?" It was read by the Secretary.

Virgil P. Gibney, A.M., M.D., of New York, read a paper on "Treatment and Management of Tubercular Spondylitis."

Nathan Allen, A.M., M.D., Lowell, Mass., presented a paper on "Physical Culture in Amherst College." He favored gymnastics over feats of ball, rowing, etc., which only developed certain muscles. It was read by the Secretary.

The following new members were elected: J. G. Sloane, A.M., M.D., Monongahela City; M. H. Borland, A.M., M.D., Pittsburg; C. H. von Klein, A.M., M.D., Dayton, Ohio; A. B. Threasher, A.M., M.D., Cincinnati; Phillip Zenner, A.M., M.D., Cincinnati; Richard Hogsden, A.M., M.D., Abington, N. Y.; W. B. Canfield, A.M., M.D., Baltimore; E. Wing, A.M., M.D., Chicago; L. Curtis, A.M., M.D., Chicago; R. I. Buntley, A.M., M.D., Pittsburg; S. D. Jennings, A.M., M.D., Allegheny; W. D. Kearns, A.M., M.D., Pittsburg; John Dubb, A.M., M.D., Pittsburg; N. J. R. Klein, A.M., M.D., Greensburg, Pa.; W. C. Shaw A.M., M.D., Pittsburg; J. C. Connell, A.M., M.D., Pittsburg.

N. S. Davis, M.D., LL.D., Chicago, was elected an honorary member.

The Nominating Committee reported the following names:

*President*—L. P. Bush, A.M., M.D., Wilmington, Delaware.

*Vice-Presidents*—R. L. Sibbett, A.M., M.D., Carlisle, Pa.; S. J. Jones, A.M., M.D., Chicago; P. S. Connor, A.M., M.D., Cincinnati; V. P. Gibney, A.M., M.D., New York.

*Secretary and Treasurer*—R. J. Dunglison, A.M., M.D., Philadelphia.

*Assistant Secretary*—Charles McIntyre, Jr., A.M., M.D., Easton, Pa.

The next annual meeting will be held in Washington, D. C., on September 2 and 3, 1887.

